# Binary IS Typing for *Staphylococcus aureus*

**DOI:** 10.1371/journal.pone.0013671

**Published:** 2010-10-27

**Authors:** Andries E. Budding, Christina M. J. E. Vandenbroucke-Grauls, Damian C. Melles, Engeline van Duijkeren, Jan A. Kluytmans, Paul H. M. Savelkoul

**Affiliations:** 1 Medical Microbiology and Infection Control, VU University Medical Center, Amsterdam, The Netherlands; 2 Department of Medical Microbiology, Erasmus Medical Center, Rotterdam, The Netherlands; 3 Department of Infectious Diseases and Immunology, Faculty of Veterinary Medicine, Utrecht University, Utrecht, The Netherlands; 4 Laboratory for Microbiology and Infection Control, Amphia Hospital, Breda, The Netherlands; University of Hyderabad, India

## Abstract

**Background:**

We present an easily applicable test for rapid binary typing of *Staphylococcus aureus*: binary interspace (IS) typing. This test is a further development of a previously described molecular typing technique that is based on length polymorphisms of the 16S-23S rDNA interspace region of *S. aureus*.

**Methodology/Principal Findings:**

A novel approach of IS-typing was performed in which binary profiles are created. 424 human and animal derived MRSA and MSSA isolates were tested and a subset of these isolates was compared with multi locus sequence typing (MLST) and Amplified Fragment Length Polymorphism (AFLP). Binary IS typing had a high discriminatory potential and a good correlation with MLST and AFLP.

**Conclusions/Significance:**

Binary IS typing is easy to perform and binary profiles can be generated in a standardized fashion. These two features, combined with the high correlation with MLST clonal complexes, make the technique applicable for large-scale inter-laboratory molecular epidemiological comparisons.

## Introduction

Methicillin-resistant *Staphylococcus aureus* (MRSA) is a growing cause of concern world-wide, not only because of the high incidence of infections with these resistant micro-organisms in hospitals, but also because of the increasing incidence of infections with MRSA acquired in the community [1]. Control measures to halt this rise, or to lower the prevalence of MRSA have been implemented in many countries. Important components of control strategies are detection of resistant strains and tracking of transmission of strains in hospitals and the community by molecular typing.

Several different molecular typing methods are available, these have recently been reviewed by Struelens et al [2]. Each method has its own strengths and weaknesses, but no single method yet combines high discriminatory power, full inter-laboratory portability, ease of performance and low cost.

We have further developed a previously described molecular typing technique that is based on length polymorphisms of the 16S-23S rDNA interspace region (IS) of *S. aureus* [3]. *S. aureus* strains typically have 5 or 6 IS regions of different sizes. Amplification of these IS regions and gel electrophoresis of the PCR products yields banding patterns that are characteristic for individual strains. Typing methods based on these IS patterns correlate well with the more mainstream molecular typing methods [4]. We adapted IS-based typing for analysis on an automated capillary gel electrophoresis machine, translated banding patterns to binary profiles to provide inter-laboratory portability, and created a digital database. The method was tested on a collection of 424 MRSA and methicillin-susceptible *S. aureus* (MSSA) isolates of human and animal origin and compared with MLST and AFLP. Finally, performance of the method was evaluated in a real world epidemiological problem: identification of pig farming-associated MRSA isolates belonging to MLST clonal complex 398 collected throughout the Netherlands. IS typing proved so practical in this last test that we developed a simple version of the test, to make it applicable in laboratories with no capillary gel electrophoresis facilities.

## Results

### *In silico* analysis

Unique band patterns were obtained with virtual PCR performed on the whole genome sequences of the two reference strains. The patterns were clearly distinguishable from each other. *In vitro* analysis of the cultured reference strains with the automatic sequencer as well as with agarose gel, showed banding patterns identical to the predicted *in silico* banding patterns. Some vague bands of less than 10% intensity were present. According to protocol, these bands were excluded from the analysis.

### Patterns obtained with capillary gel electrophoresis

**Figure 1:**
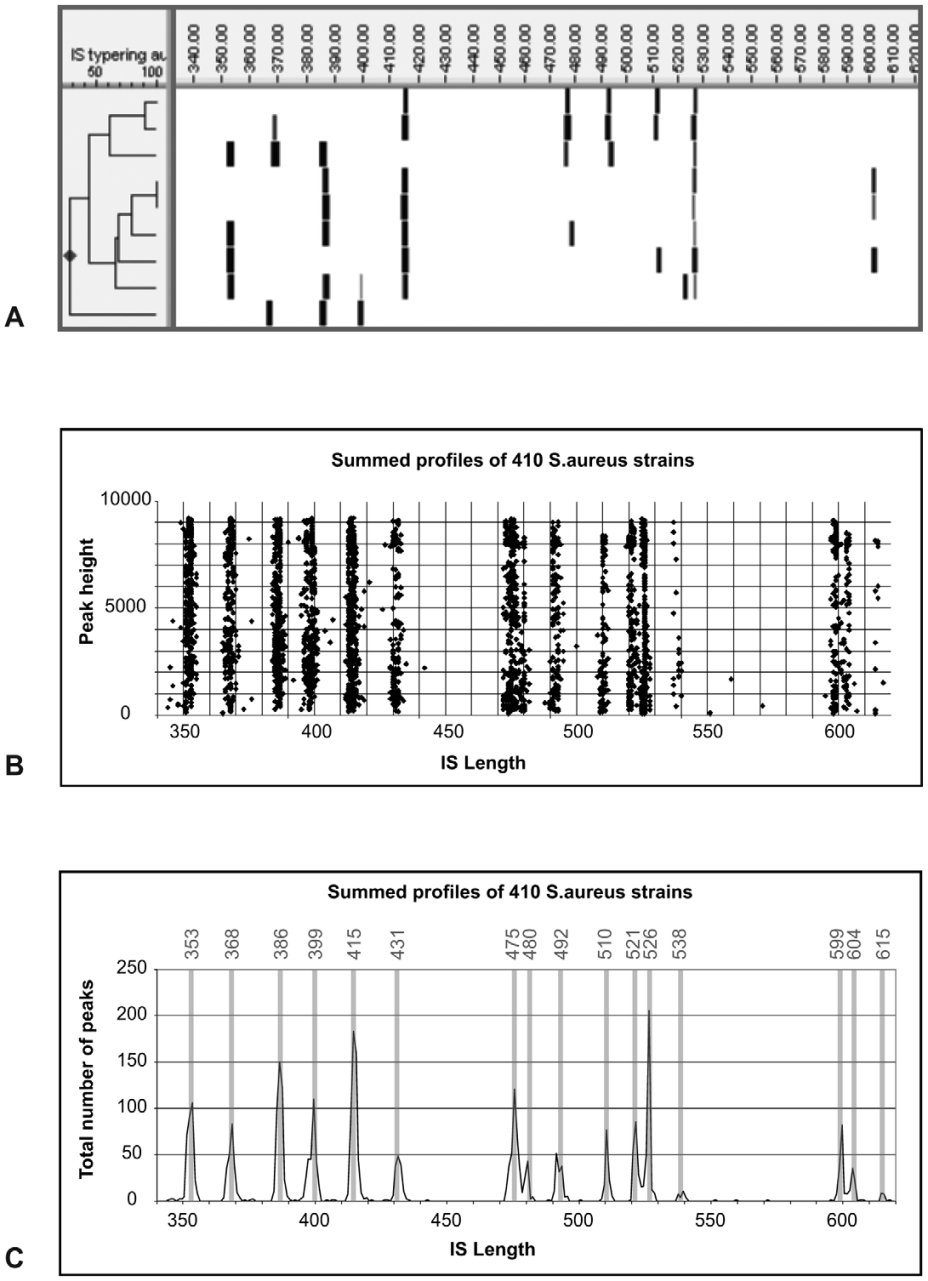
Common IS fragments in *S. aureus*. **A**: Representation of IS profiles of 9 strains of *S. aureus*. In this subset, 11 of 16 common bands are represented. **B:** Superposition of all 424 *S. aureus* profiles. Every data point represents a peak (>10% of average peak height within a profile). X-axis represents fragment length, Y-axis represents peak height in relative fluorescence units (RFU). The 16 distinct fragment lengths are clearly visible in this representation. **C:** Total number of peaks for all fragment lengths. The 16 common fragment lengths are marked by the grey lines. Not all fragment lengths are equally common, especially fragments of 538 and 615 nt length are rare. Peaks of which <10 representatives were found were not included in the set of 16 common peaks.

Capillary gel electrophoresis yielded clear patterns (figure 1A). Bands showed some variation in intensity, but were clearly distinct from background noise. Among the 424 isolates tested, 134 unique patterns were observed. Every pattern was composed of three to seven fragments of different length. Over all different patterns, the total number of different fragment lengths that we found was 16. This became especially apparent when all profiles were superimposed over one another as shown in figure 1B and C. Each band pattern could thus be assigned a binary code of 16 digits reflecting presence or absence of each defined band category. We calculated discriminatory potential of this binary typing system based on presence and absence of the 16 common bands. To this end, we assessed the number of peaks present in each profile. This number was normally distributed around five, with three and seven bands as minimum and maximum, respectively. The theoretical discriminatory potential is equal to the number of possible band patterns, which can be calculated by the sum of possible combinations of 3, 4, 5, 6 or 7 bands  = 16!/13!+16!/12!+16!/11!+16!/10!+16!/9!≈64×10^6^. To evaluate the *in vitro* discriminatory power of binary IS-typing, we compared it to MLST and AFLP in a sub selection of 89 isolates.

### Comparison with MLST and AFLP

To compare IS-typing to MLST and AFLP, a cluster analysis based on IS-typing was performed on a sub selection of 89 *S. aureus* isolates that had previously been assigned 30 unique multi locus sequence types [5]. Of these isolates, 79 could be clustered into 11 MLST clonal complexes. Ten isolates were not classifiable in this sense. AFLP analysis identified 6 major *S. aureus* lineages. The major AFLP clusters could be subdivided in 17 subclusters. AFLP major clusters 2, 3, 4, 5 and 6 correspond with MLST clonal complex 30, 45, 22, 121 and 398, respectively. Apparently, these clusters are genetically very homogeneous, but significant genetic heterogeneity could still be found with AFLP. AFLP cluster 1 was very diverse and comprised six MLST clonal complexes.

**Figure 2:**
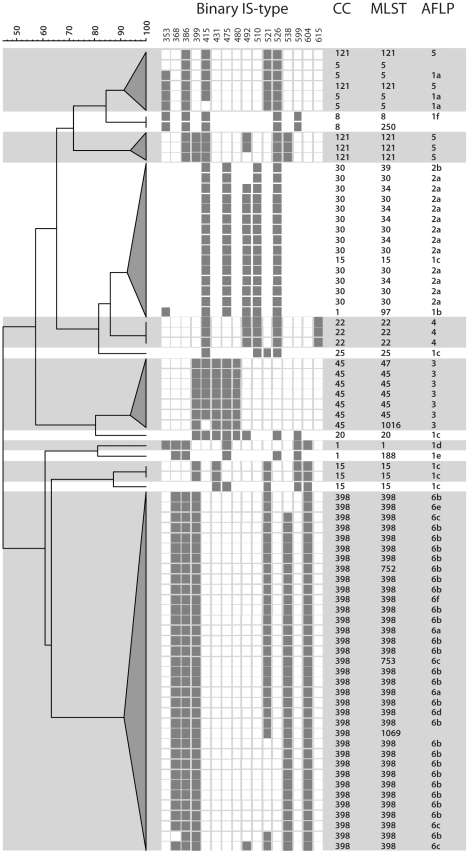
Correlation of digital IS profiles with MLST and AFLP. Thirty-three unique IS types were found belonging to 13 clusters. These clusters were defined as strains with identical binary profiles (connected by a vertical line) or as profiles differing no more than one digit from the most common profile within that group (connected by grey triangles).

In this analysis 33 unique IS types were found belonging to 13 clusters. These clusters were defined as isolates with identical binary profiles or with profiles differing no more than one digit from the most common profile within that group. IS clustering was found to have a high correlation with MLST clonal complex clustering and with major AFLP clusters (figure 2). Of the 79 isolates, correlation between CC and IS-typing was not consistent in four isolates, of CC types 1, 15 and 121. CC types 1 and 15 both fell within the genetically heterogeneous AFLP cluster 1.

### Cluster analysis of binary IS typing data

**Figure 3:**
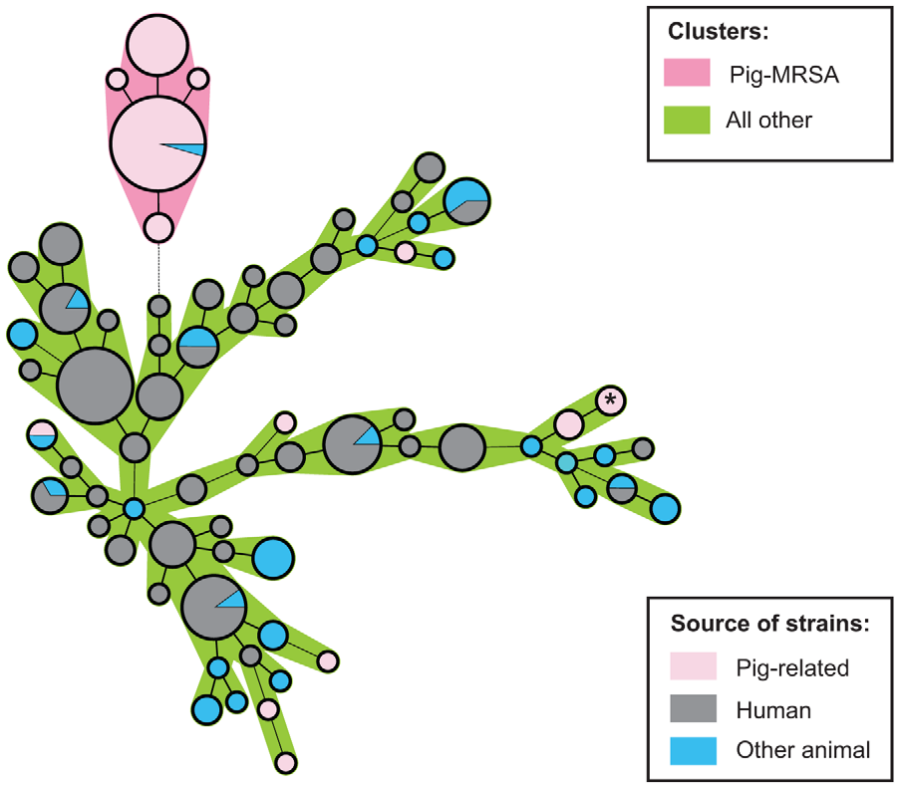
Minimal spanning tree of *S.aureus* isolates of human and animal origin. Nodes represent binary IS types. Node colour corresponds to the source of the strains, pig farming-associated, human or other animal. Node size corresponds to the number of strains of identical IS type within that node. When isolates of different sources have identical profiles, the node is depicted as a pie chart, the size of individual parts indicating relative abundance of different sources in that node. The pig farming-associated MRSA isolates (in pink) clearly form a distinct cluster. Only one of the pig farming-associated MRSA isolates (with ST 9) fell outside of this cluster and instead clustered with MSSA isolates obtained from pigs. This strain is marked with an asterisk (*) in the figure. Besides the pig farming-associated MRSA isolates, not many IS types are found in both humans and animals. Within the central node of the pig farming-associated MRSA cluster, one isolate derived from another animal can be found. This is the MSSA isolated from a monkey.

Cluster analysis of the binary coded IS typing profiles showed that almost all pig-farming-associated isolates formed one large homogenous cluster very distinct from all other isolates (figure 3). It consisted of a main group of identical types and a few small subgroups, which differed from the main cluster and from each other by no more than one band. Typically, pig-farming associated MRSA isolates were characterized by the presence of fragments of length 369, 386, 399, 520, 539 and 601nt. One pig farming-associated MRSA isolate fell entirely outside the cluster, but clustered with a number of MSSA isolates obtained from pigs. This was the isolate of the aberrant MLST 9. Remarkably, one MSSA derived from a monkey clustered with the pig farming-associated MRSA isolates. Within the isolates originating from animals, only small clusters were observed, one of which consisted of three porcine MSSA isolates and the pig farming-associated MRSA strain with MLST 9. No clear association was found between *S. aureus* IS type and host animal species. Beside the pig farming-associated MRSA cluster, only 7 of 33 animal IS types were found among the IS types found in humans. These strains mainly derived from cats (seven of ten feline strains had an IS type that was also found in humans). Thus, spread of *S. aureus* between humans and animals is not only common between humans and pigs, but also between humans and cats.

### Agarose protocol

With the agarose protocol, optimised for minimal effort, machinery and time, discrimination of all fragment lengths was not possible. Discrimination of pig farming-associated MRSA strains from other strains, however, appeared easy and straightforward. Total time needed from cultured strain to IS pattern on agarose gel was about 4 hours.

## Discussion

The invariable presence of a set of IS fragments with differing lengths in each *S. aureus* type, offers an opportunity for simple strain-level typing for this micro-organism. Different strains of *S. aureus* harbour IS fragments of various length and number. The total number of IS fragments of different lengths found in a collection of nearly 500 strains was 16; the number of different IS fragments per strain varied between 3 and 7. Based on the presence and number of these 16 defined band classes, we translated band patterns into digital profiles. Translation was performed with an automated protocol without manual interference. This eliminated subjective interpretation of band patterns.

Clustering of strains based on the resulting digital IS-profiles showed a very high correlation to MLST and AFLP based clustering. For two of the four strains where we found no correlation between MLST, AFLP and digital IS-profiles – of CC type 1 and 15 – it is known that there is no correlation between MLST and pulsed field gel electrophoresis (PFGE) [5]. For the strains that could not be classified into clonal complexes, cluster partitioning was fully consistent between IS-typing, MLST and AFLP (data not shown). The good correlation between MLST, AFLP and IS-typing confirmed the value of digital IS-typing.

Conventional slab-gel IS typing for *S. aureus* has been described before [3], [6], [7], [8], and when we corrected for primer positions, we found that ten of the sixteen characteristic IS fragments that we found have previously been described by Gurtler et al [9]. Conventional IS-typing has been reported to be well suited for inter-laboratory comparison of data [6], and IS profiles have been shown to be stable over time [10]. Stability of profiles was confirmed by our finding that IS-profiles of pig-farming associated strains were highly conserved in strains collected from many different locations across the Netherlands.

Agreement of conventional IS-typing with AFLP and PFGE has previously been reported to be good, albeit with somewhat less discriminatory power [4], [5], [11]. This implies that IS-typing may be best suited for determining deeper evolutionary relationships, a notion that is supported by our comparison with MLST and AFLP.

As an example of the application of IS typing, we have used this technique to quickly analyze pig farming-associated MRSA strains collected from different regions in The Netherlands. Although initially thought to be a problem confined mainly to the Netherlands, several reports now illustrate that spread of pig farming-associated MRSA is an international issue affecting numerous European countries and the US [12], [13], [14], [15]. This cluster analysis showed that pig-farming associated MRSA strains are clonally related and clearly distinct from other human strains or even all animal strains that we analyzed, with the exception of one MSSA strain derived from a monkey. The distance between the pig farming-associated strains and all other strains is remarkable and intriguing and warrants further investigation into the evolutionary origins of these strains. The sensitivity and specificity of IS typing as a tool for identification of pig farming-associated MRSA strains was 100% when MLST clonal cluster 398 was regarded as gold standard for identification. With the agarose gel based protocol too, identification of pig farming-associated MRSA was very straightforward.

In conclusion, we find digital IS-typing to be a simple tool for strain level typing of *S. aureus*. IS-typing correlated well with MLST and AFLP. As has been shown for MLST, the discriminatory potential of IS-typing appears lower than that of AFLP This level of discrimination, combined with the binary character of IS typing data, permitting easy inter-laboratory exchange of typing information, renders IS typing very well suited for epidemiological investigation of *S. aureus* on a national or global level.

## Methods

### Strains

The strain collection comprised 424 isolates, consisting of 125 human MRSA isolates, 144 human MSSA isolates, 42 pig farming-associated MRSA isolates obtained from humans, 10 MSSA isolates obtained from pigs, 101 other MSSA isolates obtained from other animal species and two reference MRSA strains (Mu50 and COL), whose full sequences are available in the public databases. A subset of 89 of these strains was also typed by MLST and AFLP as described by Melles et al [5]. The human strains were clinical isolates from patients admitted between 2005 and 2008 at the VU university medical center and the Erasmus university medical center; the pig farming-associated strains were obtained from the strain collection of the National Institute of Public Health and the Environment, the isolates derived from animals were clinical isolates from horses, sheep, dogs, cats, pigs, ferrets, chinchilla's and monkeys and were obtained from the Faculty of Veterinary Medicine of Utrecht University. The pig farming-associated strains were isolated from different sources across the Netherlands and were previously typed by MLST [16] and AFLP. All these pig farming-associated strains, except for one, were of MLST 398 or of the closely related type 752 or 753; the exception was a strain with MLST type 9.

### Strain culture and DNA isolation

All strains were cultured overnight on sheep blood agar (Oxoid, Cambridge, UK). A 1.0 McFarland suspension was made in tryptic soy broth (Oxoid). From this suspension, aliquots of 200 µl or 500 µl were centrifuged at 16000 g for 3 minutes in a 1 ml Eppendorf vial. The 200 µl supernatant was removed and the pellet suspended in 200 µl Tris EDTA (TE) buffer (pH 8.3). This suspension was centrifuged at 16000 g for 3 minutes. The supernatant was diluted tenfold with milliQ water and used for PCR amplification and subsequent analysis in an automated sequencer. The 500 µl aliquot of supernatant was used without further processing for PCR amplification and subsequent analysis by agarose gel electrophoresis.

### DNA amplification and analysis

For amplification of the 16S-23S rDNA spacer regions, two primers were constructed in conserved regions of 16S and 23S rDNA, respectively. The primers sequences were FirISf: CTGGATCACCTCCTTTCTAAG and DUISr1: AGGCATCCACCGTGCGCCCT. For use in capillary gel electrophoresis, a FAM labelled version of forward primer FirISf was made. The amplifications were carried out on a GeneAmp PCR system 9700 (Applied Biosystems, Foster City, CA, USA). Cycling conditions for PCR were: 94°C for 5 min, 35 cycles of 94°C for 30 s, 56°C for 45 s, 72°C for 1 min and a final extension at 72°C for 5 min. Each PCR mixture, with a final volume of 15 µl, contained 1 µl of DNA, 1x PCR buffer gold (Applied Biosystems), 1.5 mM MgCl_2_, 67 µM deoxynucleoside triphosphate (Applied Biosystems), 0.5 µl of 1.25% bovine serum albumin, 0.5 U of AmpliTaq Gold (Applied Biosystems) and 1 µM of each primer.

PCR products were analyzed either on a fully automated sequencer or by agarose gel electrophoresis. For processing on the automated sequencer, labelled PCR products were purified using the Qiagen PCR purification kit (Qiagen, Hilden, Germany) according to the instructions supplied by the manufacturer. Subsequently, 5 µl of purified PCR products were mixed with 19.9 µl formamide and 0.1 µl MAPmarker 1000 ROX-labelled size marker (BioVentures, Murfreesboro, TN, USA). DNA fragment electrophoresis was performed on an ABI Prism 3130XL Genetic Analyzer. For agarose gel electrophoresis, the PCR products (10 µl) were mixed with 1 µl loading buffer, and separated on a 1.5% agarose gel. Electrophoresis was performed at 90 V during 1.5 hours.

### Data analysis

Data were further analysed with the BioNumerics software package (Applied Maths, Sint-Martens-Latem, Belgium). Only bands with a peak height of more than 10% of the average peak height within a profile were included in analyses. Binary profiles were created for all strains based upon the presence of 16 IS fragments of distinct lengths. These fragment lengths were 353, 368, 386, 399, 415, 431, 475, 480, 492, 510, 521, 526, 538, 599, 604 and 615 nucleotides (nt). Bands were included in one of sixteen defined band classes if deviation from the defined length of that band class was no more than +/− 2nt. All identification and classification of bands was automated according to abovementioned parameters. No manual adjustments were made afterwards.

Cluster analysis was performed on binary profiles with the Dice binary similarity coefficient in combination with the Unweighted Pair Group Method with Arithmatic mean (UPGMA). Minimum spanning trees were created from binary profiles with the BioNumerics software package (Applied Maths).

Agarose gel band patterns were digitalized using the Gel Doc XR system (BioRad, Hercules, CA, USA) and interpreted visually.
